# Blockade of dengue virus transmission from viremic blood to *Aedes aegypti* mosquitoes using human monoclonal antibodies

**DOI:** 10.1371/journal.pntd.0007142

**Published:** 2019-11-01

**Authors:** Trung Tuan Vu, Hannah Clapham, Van Thi Thuy Huynh, Long Vo Thi, Dui Le Thi, Nhu Tuyet Vu, Giang Thi Nguyen, Trang Thi Xuan Huynh, Kien Thi Hue Duong, Vi Thuy Tran, Huy Le Anh Huynh, Duyen Thi Le Huynh, Thuy Le Phuong Huynh, Thuy Thi Van Nguyen, Nguyet Minh Nguyen, Tai Thi Hue Luong, Nguyen Thanh Phong, Chau Van Vinh Nguyen, Gerald Gough, Bridget Wills, Lauren B. Carrington, Cameron P. Simmons

**Affiliations:** 1 Oxford University Clinical Research Unit, District 5, Ho Chi Minh City, Vietnam; 2 Centre for Tropical Medicine and Global Health, University of Oxford, Headington, Oxford, United Kingdom; 3 Hospital for Tropical Diseases, District 5, Ho Chi Minh City, Vietnam; 4 Biopharm Discovery, GlaxoSmithKline, Stevenage, United Kingdom; 5 Institute for Vector-Borne Disease, Monash University, Clayton, Australia; Oregon Health and Science University, UNITED STATES

## Abstract

**Background:**

Dengue is the most prevalent arboviral disease of humans. Virus neutralizing antibodies are likely to be critical for clinical immunity after vaccination or natural infection. A number of human monoclonal antibodies (mAbs) have previously been characterized as able to neutralize the infectivity of dengue virus (DENV) for mammalian cells in cell-culture systems.

**Methodology/Principle findings:**

We tested the capacity of 12 human mAbs, each of which had previously been shown to neutralize DENV in cell-culture systems, to abrogate the infectiousness of dengue patient viremic blood for mosquitoes. Seven of the twelve mAbs (1F4, 14c10, 2D22, 1L12, 5J7, 747(4)B7, 753(3)C10), almost all of which target quaternary epitopes, inhibited DENV infection of *Ae*. *aegypti*. The mAbs 14c10, 747(4)B7 and 753(3)C10 could all inhibit transmission of DENV in low microgram per mL concentrations. An Fc-disabled variant of 14c10 was as potent as its parent mAb.

**Conclusions/Significance:**

The results demonstrate that mAbs can neutralize infectious DENV derived from infected human cells, in the matrix of human blood. Coupled with previous evidence of their ability to prevent DENV infection of mammalian cells, such mAbs could be considered attractive antibody classes to elicit with dengue vaccines, or alternatively, for consideration as therapeutic candidates.

## Author summary

Dengue is the most prevalent arboviral disease affecting humans. There are no therapeutics for the disease. We developed a novel virus neutralization assay, employing *Ae*. *aegypti* mosquitoes and viremic blood from dengue patients, to examine the virus-neutralizing potency of 12 human-derived monoclonal antibodies (mAbs) that had previously been shown to neutralize DENV in cell-culture systems. Five of the twelve mAbs failed to block dengue virus infections of mosquitoes using our assay. The remaining seven mAbs neutralized at least one serotype of dengue virus. The results demonstrate that some mAbs were functional and potently neutralized DENV in the complex matrix of viremic human blood. These findings advance the understanding of the types of antibodies that would be desirable to elicit using a dengue vaccine or to apply for acute therapy.

## Introduction

DENV infections are highly prevalent in the tropical and subtropical world [1]. Following a primary DENV infection it is widely accepted that an individual develops long-lived clinical immunity to the infecting serotype but not to other serotypes. DENV infection can be subclinical, or result in a febrile syndrome that in a small percentage of patients is complicated by vascular leakage, thrombocytopenia and altered hemostasis, usually between the fourth and sixth days of illness [2]. The risk of clinically important complications is higher when an individual is infected for a second time with a different DENV serotype from the first [3]. In this situation, cross-reactive antibodies are hypothesized to enhance the viral infection [4].

Antibodies are central to the concepts of dengue pathogenesis and naturally-acquired or vaccine-elicited immunity. For example, the induction of virus neutralizing antibodies by vaccination is the minimum goal of most candidate dengue vaccines [5]. The only-licensed dengue vaccine, Dengvaxia, was developed on the basis that it could elicit antibodies that neutralized all four serotypes of DENV *in vitro* [6]. Unfortunately, the vaccine mediated an increased risk of symptomatic dengue in vaccine recipients who were seronegative at baseline [7]. Despite the agreed centrality of antibodies to dengue pathogenesis, laboratory correlates of immunity (or disease enhancement risk) have not been tightly defined or standardized. There is; however, a general consensus that increasingly higher serum concentrations of neutralizing antibodies are associated with reduced risk of dengue [8, 9].

The ability to generate and characterize human mAbs from dengue immune donors has provided insights into the type of antibodies that might be desirable to elicit via immunization, or alternatively, to use as therapeutic agents. Most prominent are those human mAbs identified as being able to neutralize DENV infectivity for mammalian cells [10–16]. Some of these neutralizing mAbs bind to viral envelope proteins that have quaternary structures [17–20].

Here we developed a viremic blood neutralization assay (ViBNA), in which blood from dengue patients was spiked with mAbs, and was then used to feed *Ae*. *aegypti* mosquitoes. We used this assay to characterize a panel of 12 anti-DENV human mAbs with different serotype specificities and epitope binding characteristics. We more deeply characterized a small panel of mAbs for their effective concentrations (EC_50_) that neutralized DENV. Collectively, the results define the potency of these mAbs in the complex matrix of human blood, and against populations of DENV that have replicated in human tissues, which can inform dengue vaccine development and therapeutic approaches.

## Methods

### Viremic blood neutralization assay (ViBNA)

Viremic blood was drawn from NS1-positive dengue patients and spiked with various concentrations of individual mAbs. The blood-antibody mixture was incubated at 37°C for 30 minutes to allow antibodies to bind to DENV. Controls were prepared in parallel; the positive control for blocking DENV transmission was inactivated hyper-immune plasma, created by pooling early convalescent plasma samples from 119 dengue cases. The negative control was 0.9% saline. Both controls were spiked into viremic blood at a 1:9 dilution. The blood-antibody mixture of between 100–300 μL in volume was then maintained at 37°C in artificial membrane feeders, attached to a circulating warm water system. Mosquitoes were allowed to feed on the antibody-blood mixture for maximum 1 hr. Between 10–50 wild-type *Ae*. *aegypti* were allowed to feed on each antibody-blood preparation, engorged mosquitoes selected and maintained for 5–7 days. Those mosquitoes surviving the incubation period were killed, homogenized, and tested for the presence of DENV RNA using qualitative RT-PCR procedures described elsewhere [21]. Mosquitoes were scored as either infected or uninfected with DENV.

### Dengue patient cohorts and diagnostic tests

Eligible patients who were admitted to inpatient wards at the Hospital for Tropical Diseases in HCMC were enrolled between April 2013 and July 2017. The inclusion criteria were: age ≥15 years, <96 hours of fever history and a positive NS1 rapid test to confirm DENV infection. There were no exclusion criteria. A single 3–5 mL venous blood sample, to be used for mosquito feeding, was drawn from each participant on the day of enrolment, or occasionally, the following day. RT-PCR was performed to quantify plasma viremia levels following previously described methods [22]. Panbio’s indirect IgG ELISA was also employed to measure the presence of DENV plasma IgG.

### Ethics statement

This study was approved by the Ethics Committee of the Hospital for Tropical Diseases, Vietnam (CS/ND/12/16, CS/ND/16/27) and the Oxford University Tropical Research Ethics Committee, UK (OxTREC 30–12, OxTREC 45–16). All patients provided written informed consent to participate in the study.

### *Ae*. *aegypti* mosquitoes

We used 3–5 days-old F3 laboratory-reared *Ae*. *aegypti* mosquitoes in these experiments. All mosquitoes were derived from field-sourced materials (eggs or larvae) collected in HCMC. Mosquitoes were maintained at 28°C, with a 12:12 light:dark cycle and 80% relative humidity. Mosquito colonies were blood-fed directly on healthy volunteers and had access to 10% sucrose *ad libitum*.

#### Human monoclonal antibodies

Clone names and the previously reported DENV specificity and neutralization potency of the 12 human mAbs used in this current study are described in S1 Table. The mAb 14c10 was obtained in two forms; wildtype and LALA. The LALA modification, which results in point mutations (L234A, L235A) at the Fc of 14c10, abrogates 14c10 binding to Fc receptors.

### Data and statistical analysis

We excluded blood meals from the analysis for any of the following reasons: 1) non-infectious, i.e. failed to infect mosquitoes in the negative control (saline) group; 2) all mosquitoes in either the negative or positive control died before harvesting; 3) the number of harvested mosquitoes from a single cohort was less than four. Due to the nested nature of the data, i.e. mosquitoes fed on each preparation of blood of the same dengue patient, a marginal logistic regression model was used to evaluate the neutralization capacity of any given mAb, relative to the outcome of the negative control used for that blood sample. The model was multivariable, adjusting for the covariate of plasma viremia. A *p* value of less than 0.001, adjusted for multiple-comparisons by the Bonferroni correction method, was considered statistically significant.

After antibody treatment, the reduction in percentage of mosquitoes with DENV infection was calculated for each patient blood meal as follow:

$$100-\left(\frac{\text{The}\;\text{proportion}\;\text{of}\;\text{infected}\;\text{mosquitoes}\;\text{in}\;\text{the}\;\text{test}\;\text{mAb}\;\text{group}}{\text{The}\;\text{proportion}\;\text{of}\;\text{infected}\;\text{mosquitoes}\;\text{in}\;\text{the}\;\text{saline}\;\text{group}}\right)\times 100$$

The calculation for EC_50_ values (concentrations at which the reduction in percentage of mosquitoes with DENV infection is at 50%) was based on dose-response analysis using the three-parameter log-logistic function in the package drc in R [23]. The 95% CI was derived based on delta method without adjustment for the nested nature of the data.

## Results

### Patient blood samples used in this study

In our patient population, DENV-1 and DENV-4 were the most prevalent serotypes. The median plasma DENV-RNAemia level varied by serotypes and was highest for DENV-1 (S1 Fig).

### Outcome of experimental spiking of viremic blood samples with human mAbs

At a concentration of 10 μg/mL, five mAbs (1C19, 1M7, 22.3, 82.11 and 87.1) were unable to neutralize the infectivity of viremic blood for *Ae*. *aegypti* (Table 1 and S2 Fig). Seven mAbs inhibited the infectivity of viremic blood for mosquitoes, as shown by p-values ≤ 0.001 (Table 1 and S2 Fig). 1F4 was modestly active against DENV-1, and 5J7 against DENV-2. The mAbs 14c10 (DENV-1 specific), 1L12 and 2D22 (both DENV-2 specific) blocked viral infection of mosquitoes at 10 μg/mL. The E-dimer epitope (EDE)-specific mAbs 747(4)B7 and 753(3)C10, both previously nominated as serotype cross-reactive, had different potency characteristics at the highest concentrations tested (3.7 μg/mL and 5 μg/mL, respectively). 753(3)C10 blocked DENV-1 and DENV-4 but did not prevent mosquitoes from acquiring DENV-2 infection. 747(4)B7 was also highly potent against DENV-1, but only partially blocked DENV-2 and DENV-4. There were an insufficient number of patients with DENV-3 infections to characterize the potency of any of these mAbs against DENV-3.

**Table 1:** Neutralization capacity of monoclonal antibodies in the viremic blood neutralization assay. Mosquitoes were fed on blood meals containing DENV infected blood and 10 μg/mL of individual mAbs, except for 747(4)B7 and 753(3)C10 at 3.7 μg/mL and 5 μg/mL, respectively. DENV infected mosquitoes were detected by RT-PCR. Odds ratios were calculated using data from each mAb with the negative saline control as the reference. *P* values were calculated using marginal logistic regression models, adjusted for the patient’s plasma viremia. The reduction in percentage of mosquito infection, compared with the negative saline control, was calculated for each blood feeding cohort. DENV = dengue virus; PC = positive control; NC = negative control.

mAb	Viral Serotype	Number of blood meals	Percentage of infected mosquitoes (Total number of mosquitoes tested)			OR (95% CI)	*P*	Reduction in percentage of mosquito infection (mean ± SE)
			PC	mAb	NC			
1C19	DENV-1	6	3 (30)	70 (30)	84 (57)	0.38 (0.13–1.13)	0.08	21.33 ± 17.57
	DENV-2	5	0 (22)	88 (25)	90 (43)	0.70 (0.20–2.38)	0.56	1.78 ± 7.08
	DENV-3	3	0 (15)	67 (15)	61 (28)	2.46 (0.51–11.81)	0.26	16.67 ± 41.67
	DENV-4	8	3 (39)	72 (39)	51 (79)	2.58 (0.89–7.51)	0.08	-70.99 ± 49.98
1F4	DENV-1	35	3 (272)	53 (294)	83 (308)	0.18 (0.10–0.34)	**<0.001**	37.24 ± 7.56
	DENV-2	6	0 (51)	95 (44)	95 (57)	1.23 (0.31–4.90)	0.77	-4.44 ± 4.68
	DENV-3	2	7 (15)	100 (15)	65 (20)	NA^a^	NA^a^	-62.50 ±37.50
	DENV-4	17	5 (131)	72 (123)	77 (149)	0.78 (0.41–1.49)	0.45	0.33 ± 9.28
1M7	DENV-1	4	5 (20)	63 (19)	78 (37)	0.28 (0.10–0.80)	0.02	31.78 ± 22.99
	DENV-2	5	0 (24)	96 (23)	95 (41)	1.07 (0.19–6.19)	0.93	-4.44 ± 7.54
	DENV-3	2	0 (10)	90 (10)	80 (20)	2.26 (0.48–10.68)	0.30	-12.50 ± 12.50
	DENV-4	8	5 (39)	69 (39)	53 (75)	1.96 (0.55–7.01)	0.30	-77.40 ± 52.56
22.3	DENV-1	6	0 (75)	67 (30)	82 (57)	0.38 (0.10–1.41)	0.15	23.93 ± 20.82
	DENV-2	0	NA	NA	NA	NA	NA	NA
	DENV-3	1	0 (5)	80 (5)	80 (10)	NA^a^	NA^a^	NA
	DENV-4	8	3 (65)	38 (40)	45 (76)	0.71 (0.28–1.76)	0.84	5.72 ± 23.21
5J7	DENV-1	24	2 (188)	80 (208)	81 (217)	0.89 (0.50–1.57)	0.68	8.92 ± 8.99
	DENV-2	4	0 (39)	85 (39)	93 (45)	0.46 (0.38–0.54)	**<0.001**	6.67 ± 4.71
	DENV-3	4	4 (25)	52 (25)	58 (38)	0.70 (0.18–2.70)	0.60	27.50 ± 35.15
	DENV-4	11	5 (89)	73 (93)	75 (123)	0.70 (0.39–1.28)	0.25	10.94 ± 12.31
82.11	DENV-1	8	1 (85)	78 (40)	86 (77)	0.52 (0.23–1.17)	0.12	11.28 ± 9.07
	DENV-2	5	0 (24)	88 (25)	95 (41)	0.34 (0.07–1.82)	0.21	4.00 ± 11.27
	DENV-3	3	0 (15)	53 (15)	61 (28)	0.76 (0.38–1.52)	0.44	-11.67 ± 25.22
	DENV-4	9	4 (83)	36 (45)	44 (86)	0.69 (0.35–1.36)	0.29	12.69 ± 20.47
87.1	DENV-1	6	2 (60)	67 (30)	87 (60)	0.15 (0.03–0.79)	0.03	29.63 ± 19.17
	DENV-2	5	0 (24)	96 (25)	95 (41)	1.18 (0.36–3.91)	0.79	-3.56 ± 2.29
	DENV-3	3	0 (15)	53 (15)	61 (28)	0.74 (0.17–3.15)	0.68	33.33 ± 36.32
	DENV-4	9	3 (71)	52 (44)	51 (86)	1.02 (0.52–2.00)	0.94	6.78 ± 19.62
1L12	DENV-1	25	3 (196)	81 (214)	81 (223)	1.00 (0.55–1.83)	0.99	0.97 ± 7.38
	DENV-2	9	0 (62)	8 (72)	92 (89)	0.01 (0.00–0.02)	**<0.001**	89.67 ± 3.78
	DENV-3	2	7 (15)	93 (15)	65 (20)	8.56 (0.46–158)	0.15	-50.00 ±50.00
	DENV-4	11	5 (85)	71 (91)	72 (125)	0.83 (0.34–1.99)	0.68	-3.55 ±11.37
2D22	DENV-1	25	2 (196)	79 (218)	81 (221)	0.84 (0.48–1.49)	0.55	2.92 ± 6.43
	DENV-2	8	0 (58)	8 (73)	95 (82)	0.01 (0.00–0.02)	**<0.001**	91.94 ± 3.74
	DENV-3	2	7 (15)	100 (15)	65 (20)	NA^a^	NA^a^	-62.50 ± 37.50
	DENV-4	11	4 (89)	74 (92)	75 (123)	0.75 (0.42–1.34)	0.33	0.84 ± 9.06
747(4)B7	DENV-1	22	3 (181)	1 (197)	82 (190)	0.00 (0.00–0.01)	**<0.001**	98.31 ± 0.93
	DENV-2	4	0 (39)	58 (40)	93 (45)	0.09 (0.03–0.23)	**<0.001**	37.92 ± 11.00
	DENV-3	1	10 (10)	0 (10)	50 (10)	NA^a^	NA^a^	NA
	DENV-4	9	5 (79)	28 (83)	82 (103)	0.07 (0.03–0.17)	**<0.001**	69.37 ± 7.26
753(3)C10	DENV-1	23	3 (189)	4 (204)	83 (200)	0.01 (0.00–0.02)	**<0.001**	94.87 ± 1.77
	DENV-2	4	0 (39)	80 (40)	93 (45)	0.30 (0.12–0.75)	0.01	12.08 ± 7.92
	DENV-3	1	10 (10)	80 (10)	50 (10)	NA^a^	NA^a^	NA
	DENV-4	9	5 (79)	2 (89)	82 (103)	0.00 (0.00–0.02)	**<0.001**	97.78 ± 1.47
14c10	DENV-1	23	3 (189)	7 (214)	82 (200)	0.01 (0.00–0.03)	**<0.001**	92.80 ± 2.51
	DENV-2	4	0 (39)	98 (40)	93 (45)	3.51 (0.16–74.7)	0.42	-10.00 ± 9.53
	DENV-3	1	10 (10)	90 (10)	50 (10)	NA^a^	NA^a^	NA
	DENV-4	9	5 (79)	79 (87)	82 (103)	0.83 (0.23–3.02)	0.77	-4.41 ±14.30

^a^Numbers of blood meals were too few to analyse

Unlike the other five serotype-specific mAbs, 747(4)B7 and 753(3)C10 were previously characterized as cross-reactive antibodies, so we hypothesized that their neutralization capacity might be influenced by the presence of pre-existing patient-derived antibodies, such as those binding to the E fusion loop region that compete and block access to the EDE quaternary epitope. We therefore tested for the presence of patient-derived IgG to the DENV virion (assessed by Panbio IgG indirect ELISA) in the acute viremic blood samples that were used to feed mosquitoes. Upon analysis, we could not distinguish between an IgG competition effect and a serotype effect (S2 Table) because DENV-2 and DENV-4 viremic blood meals were also typically associated with the presence of IgG to the DENV virion, i.e. almost all viremic blood samples from patients with DENV-2 and DENV-4 had detectable DENV-reactive IgG (S3 Table). Collectively, we identified several mAbs capable of reducing, or abrogating, the infectivity of dengue patients’ blood for mosquitoes.

### Effective concentrations (EC_50_) of a subset of neutralizing mAbs

**Fig 1:**
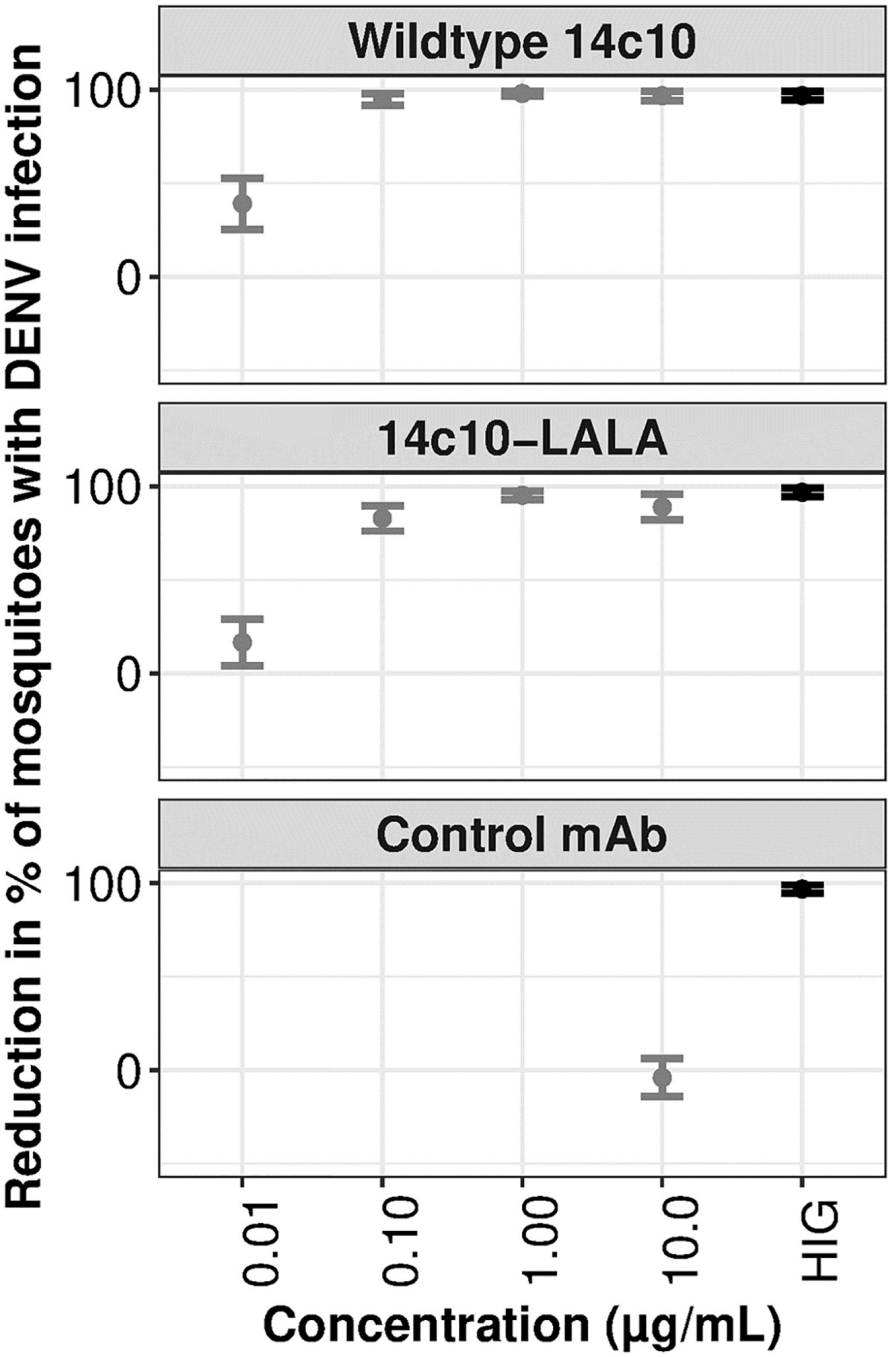
Both 14c10 variants neutralize DENV-1 in low microgram per mL concentrations. The same blood samples were used between mAbs. The y-axis value of 100 means the mAb blocks DENV infection of mosquitoes completely, relative to the negative control. The isotype control mAb binds to respiratory syncytial virus. Means and standard errors of more than two replicates are shown. The positive control (hyper-immune globulin (HIG)) is in black while tested mAbs are in gray.

Because there were relatively fewer patients with DENV-2 infections at the time of the study, we were unable to enroll enough patients with this serotype to estimate the EC_50_ of the DENV-2 specific mAbs (2D22, 1L12). Using the ViBNA, we found wildtype 14c10 neutralized DENV-1 at an EC_50_ of 0.013 μg/mL (95%CI 0.005–0.020). The EC_50_ concentration of 14c10 was also independent of Fc receptor engagement as the Fc disabled variant 14c10-LALA was just as potent as the parent mAb when they were tested on identical blood meals (Fig 1). The EC_50_ concentration for 14c10-LALA against DENV-1 was at 0.026 μg/mL (95%CI 0.006–0.045). The EC_50_ for 747(4)B7 against DENV-1 and DENV-4 were at 0.056 μg/mL (95% CI not measurable—0.179), and 1.104 μg/mL (not measurable—13.921), respectively (S3 Fig). The EC_50_ for 753(3)C10 against DENV-1 and DENV-4 were at 0.643 μg/mL (95% CI not measurable—2.291), and 1.289 μg/mL (not measurable—16.433), respectively (S4 Fig).

## Discussion

Here we tested a panel of well-characterized human monoclonal antibodies (mAbs) for their capacity to block the infectivity of human viremic blood for *Ae*. *aegypti* mosquitoes. The mAbs that recognized quaternary epitopes on the virion surface were generally the most potent. An Fc-disabled variant of the DENV-1 specific mAb 14c10 was as potent as its wild-type parent, confirming that Fc receptor engagement was not necessary for virus neutralization in this model.

All of the 12 human mAbs tested in this current study have been shown to neutralize the infectivity of DENV in cell-culture systems (S1 Table). The present study extends the understanding of these mAbs by demonstrating that some were also potent at reducing the infectivity of DENV in the matrix of viremic human blood and abrogating infection of blood-feeding mosquitoes. This provides novel insights because the matrix of human blood from a dengue case is a complex environment containing virions derived from infected human cells in a milieu of plasma proteins, in a tight pH environment, and in some patients, pre-existing anti-DENV antibodies acquired from a previous infection. We hypothesize that those mAbs that are active in the ViBNA may bind DENV virions in patient blood and prevent attachment to the surface of mosquito midgut epithelial cells. We also hypothesize that the polyclonal nature of the pooled plasma, containing high levels of neutralizing DENV-reactive antibodies, could deliver “multi-hit” virus neutralization that is more potent than most mAbs.

Raut *et al* recently reported that DENV virions in human blood are more mature and more infectious for mammalian cells than virions from cultured cells [24]. This previous work, coupled with the results in the current study, highlight the stringency of virus neutralization assays using *in vivo* (human) propagated virus when compared to assays that use *in vitro* propagated virus. In essence, higher concentrations of either monoclonal or polyclonal antibody might be needed to neutralize *in vivo* (human) propagated virus because a high proportion of these virions are mature. Future investigations of antibody correlates of immunity on either natural history or vaccine studies should consider the differences between *in vivo* and *in vitro* propagated virus.

The magnitude of virus neutralization mediated by some mAbs was striking. For example, in acute viremic blood samples that contained relatively high concentrations of DENV-1 (S1 Fig), the 14c10 mAb very effectively blocked the blood’s infectiousness. As expected, this occurred independently of Fc receptor engagement because a LALA variant displayed the same levels of activity. The rationale for testing a LALA variant of 14c10 was to demonstrate that its potency would not be diminished by this mutation. This is of relevance because any antibody-based therapeutic candidate for dengue is likely to be engineered to be devoid of Fc receptor binding for safety reasons, such as mitigating the risk of Fc-mediated pathogenic pathways.

The mAbs 747(4)B7 (EDE2) and 753(3)C10 (EDE1), which recognize quaternary epitopes, were also potent neutralizers of virus infectivity, but with the caveat that their potency was lower in patient blood samples that already contained IgG to DENV virions, i.e. blood that contained anti-DENV antibodies elicited by a past infection. We could not however discriminate whether the lower potency was a serotype effect or an antibody competition effect because of extensive co-correlation between DENV-2 and DENV-4 infections and the presence of pre-existing anti-DENV antibodies. The antibody competition scenario is plausible since 747(4)B7 and 753(3)C10 mAbs target the same residues from three polypeptide segments on domain II of the envelope dimer: the *b* strand (amino acids 67–74, bearing the N67 glycan), the fusion loop and residues immediately upstream (amino acids 97–106), and the *ij* loop (amino acids 246–249). Thus we think it’s plausible that the pre-existing anti-DENV IgG in these patients (particularly antibodies binding to the fusion loop, a long-established target of serotype cross-reactive but poorly neutralizing antibodies [10, 15]), sterically interferes with the binding of 747(4)B7 and 753(3)C10 to their epitopes.

What of antibodies that were not potent in the ViBNA? We cannot conclude that these mAbs lacked virus neutralization activity, only that they lacked sufficient potency to reduce the titer of infectious virus in the patients’ blood samples (against which they were tested) to a level needed to infect mosquitoes [25]. A common feature of mAbs that had no or intermediate potency in the ViBNA was that they recognized epitopes available on the soluble envelope dimer (e.g. 22.3 and 1C19). This, and other work [20], suggests that vaccine approaches that present intact virions to the immune system, e.g. live attenuated viruses [26] or inactivated whole virion vaccines, are most likely to elicit the kind of quaternary-epitope-specific antibodies that in the ViBNA were most potent.

Our study had several limitations and sources of natural variance. The concentration of DENV naturally varied between patients. However, we could not attempt to “standardize” plasma viremia between experiments for multiple logistical reasons. One of them is that it would have undermined the integrity of the whole blood environment. Another limitation is that we could not measure the potency of all mAbs against all four serotypes of DENV because this was governed by the DENV serotype prevalence in the community during the study period. The third limitation is that the ViBNA effectively only detected virus neutralization when the mAb could reduce the infectious titer to below the mosquito infectious dose. Lastly, the ViBNA is also labor-intensive, time-consuming, and requires special facilities. All of these limit its widespread application. Despite these limitations, this study has further defined the characteristics of a panel of human mAbs. The insights into mAb potency are unique because they bring together viremic blood, and hence virus that has replicated in human tissues, with the primary mosquito vector, *Ae*. *aegypti*. Antibodies that neutralize DENV in conventional virus neutralization assays, and also the ViBNA, are the type of antibodies that might be desirable to elicit with a dengue vaccine, or to trial as therapeutic agents.

## Supporting information

S1 Fig Flow chart of the processing of viremic blood neutralization assay. Briefly, aliquots of viremic blood were spiked with mAbs. Each mAb and a viremic blood sample was used to feed a cohort of mosquitoes. Engorged mosquitoes were then collected and tested for dengue virus (DENV) RNA. Excluded data originated from cases of non-infectious blood meals or from cases where all mosquitoes within the positive or negative control cohorts died before being collected, and therefore could not be assessed. Viremic blood samples were independently assessed for DENV serotype and viremia.
(TIF)

S2 Fig An example of neutralizing and non-neutralizing monoclonal antibodies to see the profile for each of these types of antibodies. The blood samples were not the same between mAbs. At the top of each panel are the mAb clone names. While 1F4 (10 μg/mL) and 82.11 (10 μg/mL) failed to neutralize DENV of any serotypes, 2D22 (10 μg/mL) is DENV-2 specific and 753(3)C10 (5 μg/mL) is cross-reactive against DENV-1 and DENV-4. This is indicated by the y-axis values of 100, meaning the mAb neutralizes DENV, relative to the negative control. Means and standard errors of test results are calculated from three or more independent ViBNA measurements. Positive control is in black while tested mAbs are in gray.
(TIF)

S3 Fig 747(4)B7 neutralized DENV-1 and DENV-4 in low microgram per mL concentrations. Means and standard errors of more than two replicates are shown. Hyper-immune dengue virus (DENV)-reactive globulin (HIG), used as positive control, is in black while monoclonal antibodies are in gray. The y-axis value of 100 means that the mAb blocks DENV infection of mosquitoes completely, relative to the negative control.
(TIF)

S4 Fig 753(3)C10 neutralized DENV-1 and DENV-4 in low microgram per mL concentrations. Means and standard errors of more than two replicates are shown. Hyper-immune dengue virus (DENV)-reactive globulin (HIG), used as positive control, is in black while monoclonal antibodies are in gray. The y-axis value of 100 means the mAb blocks DENV infection of mosquitoes completely, relative to the negative control.
(TIF)

S5 Fig The correlation between viremia titer and the percentage of infected mosquitoes. 753(3)C10 and 82.11 were representative of effective and non-effective mAbs, respectively. Each dot represents a single blood meal spiked with the two representative mAbs (10μg/mL).
(TIF)

S1 Table Previously characterized properties of tested monoclonal antibodies. Serotype specificity, IC_50_, and types of epitopes to which these mAbs bind were previously characterized in cited references. Unlike quaternary epitope, linear epitopes consists of individual, separate E proteins. DENV = dengue virus, IC = Inhibitory concentrations.
(DOCX)

S2 Table Indistinguishable effect of patients-derived DENV-reactive IgG and DENV serotypes. Neutralization capacity of 747(4)B7 and 753(3)C10 in the viremic blood neutralization assay, with data for patients-derived DENV-reactive IgG. Mosquitoes were fed on blood meals containing DENV infected blood and 747(4)B7 and 753(3)C10 at 3.7 μg/mL and 5 μg/mL, respectively. Infected mosquitoes were then detected with the presence of DENV RNA. Odds ratios were calculated using data from each mAb with the negative saline control as the reference. *P* values were calculated using marginal logistic regression models, adjusted for the patient’s plasma viremia and the detection of patients’ DENV-reactive IgG. DENV = dengue virus; PC = positive control; NC = negative control.
(DOCX)

S3 Table DENV-2 and DENV-4 viremic blood meals are typically associated with the presence of IgG to the DENV virion. (DOCX)
