# Supplementary figures and images for: Blockade of dengue virus transmission from viremic blood to Aedes aegypti mosquitoes using human monoclonal antibodies
Source: PLoS Negl Trop Dis. 2019 Nov 1;13(11):e0007142. doi: 10.1371/journal.pntd.0007142 (PMC6853333; doi:10.1371/journal.pntd.0007142)

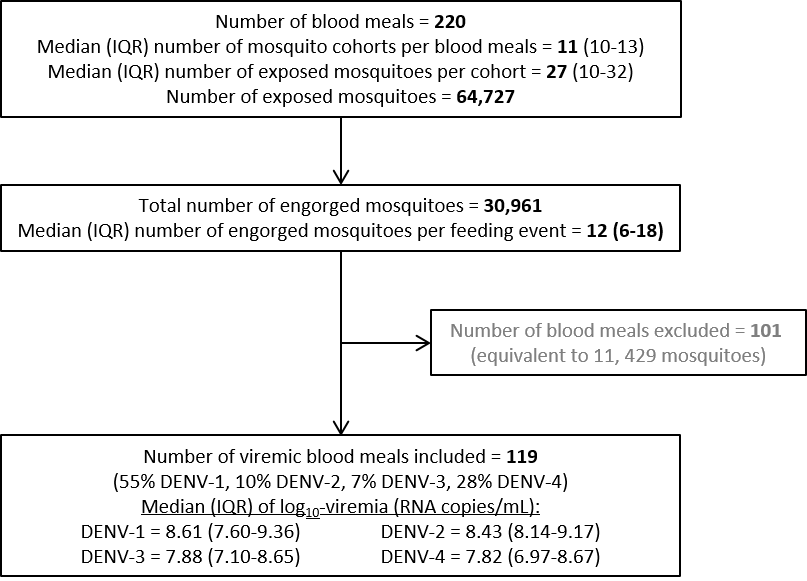

Supplement: S1 Fig — Briefly, aliquots of viremic blood were spiked with mAbs. Each mAb and a viremic blood sample was used to feed a cohort of mosquitoes. Engorged mosquitoes were then collected and tested for dengue virus (DENV) RNA. Excluded data originated from cases of non-infectious blood meals or from cases where all mosquitoes within the positive or negative control cohorts died before being collected, and therefore could not be assessed. Viremic blood samples were independently assessed for DENV serotype and viremia. (TIF) [file pntd.0007142.s001.tif]

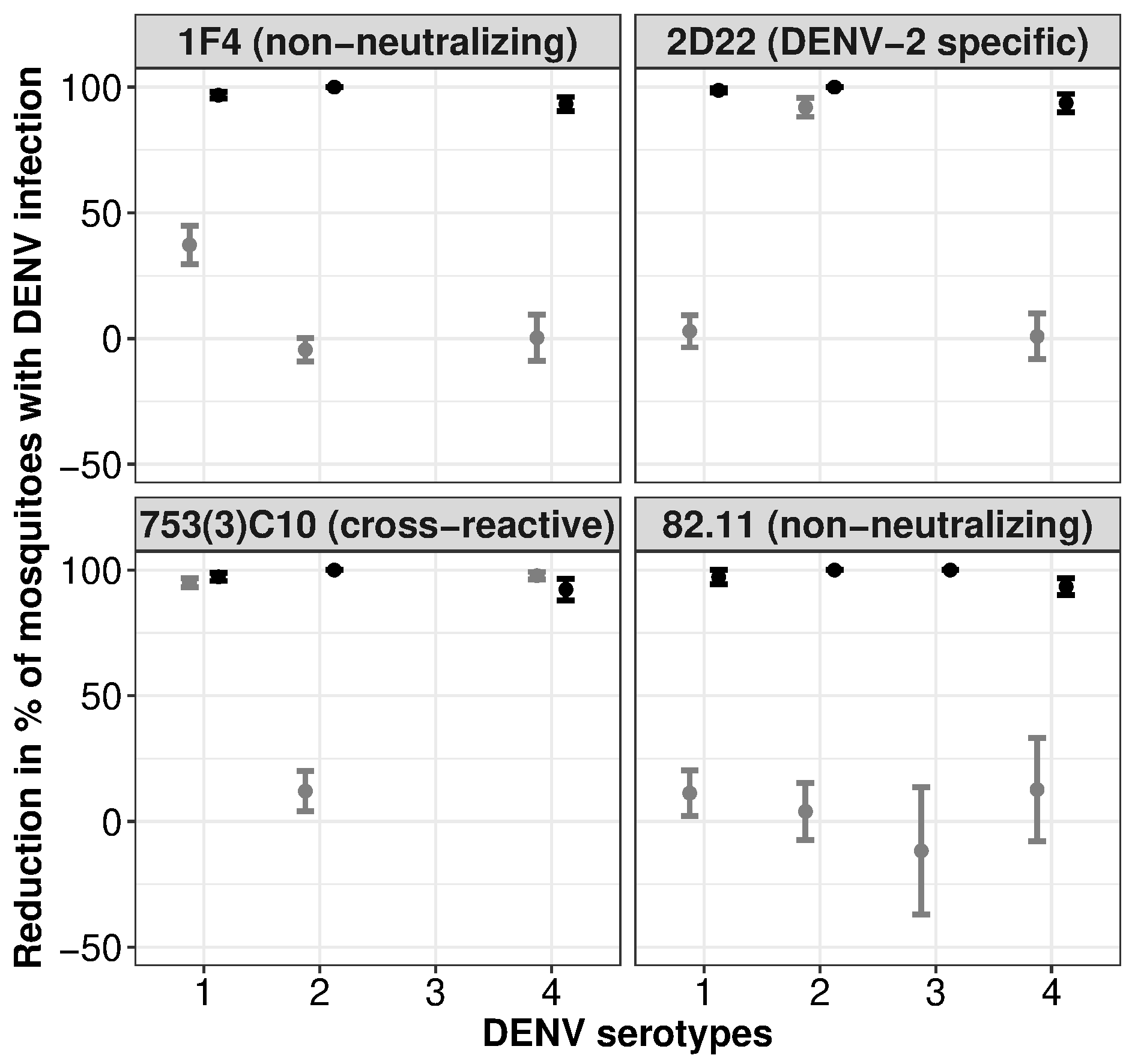

Supplement: S2 Fig — The blood samples were not the same between mAbs. At the top of each panel are the mAb clone names. While 1F4 (10 μg/mL) and 82.11 (10 μg/mL) failed to neutralize DENV of any serotypes, 2D22 (10 μg/mL) is DENV-2 specific and 753(3)C10 (5 μg/mL) is cross-reactive against DENV-1 and DENV-4. This is indicated by the y-axis values of 100, meaning the mAb neutralizes DENV, relative to the negative control. Means and standard errors of test results are calculated from three or more independent ViBNA measurements. Positive control is in black while tested mAbs are in gray. (TIF) [file pntd.0007142.s002.tif]

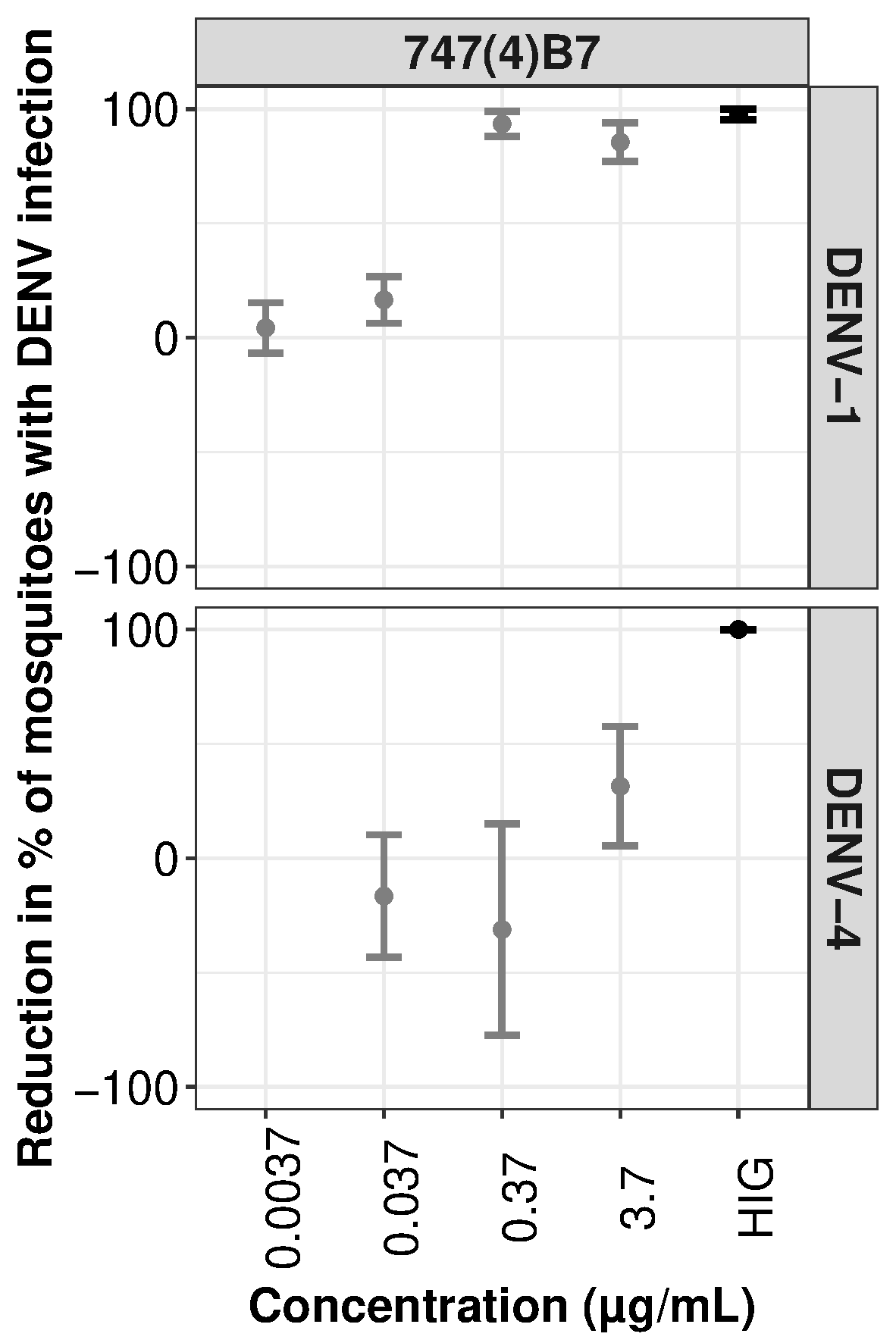

Supplement: S3 Fig — Means and standard errors of more than two replicates are shown. Hyper-immune dengue virus (DENV)-reactive globulin (HIG), used as positive control, is in black while monoclonal antibodies are in gray. The y-axis value of 100 means that the mAb blocks DENV infection of mosquitoes completely, relative to the negative control. (TIF) [file pntd.0007142.s003.tif]

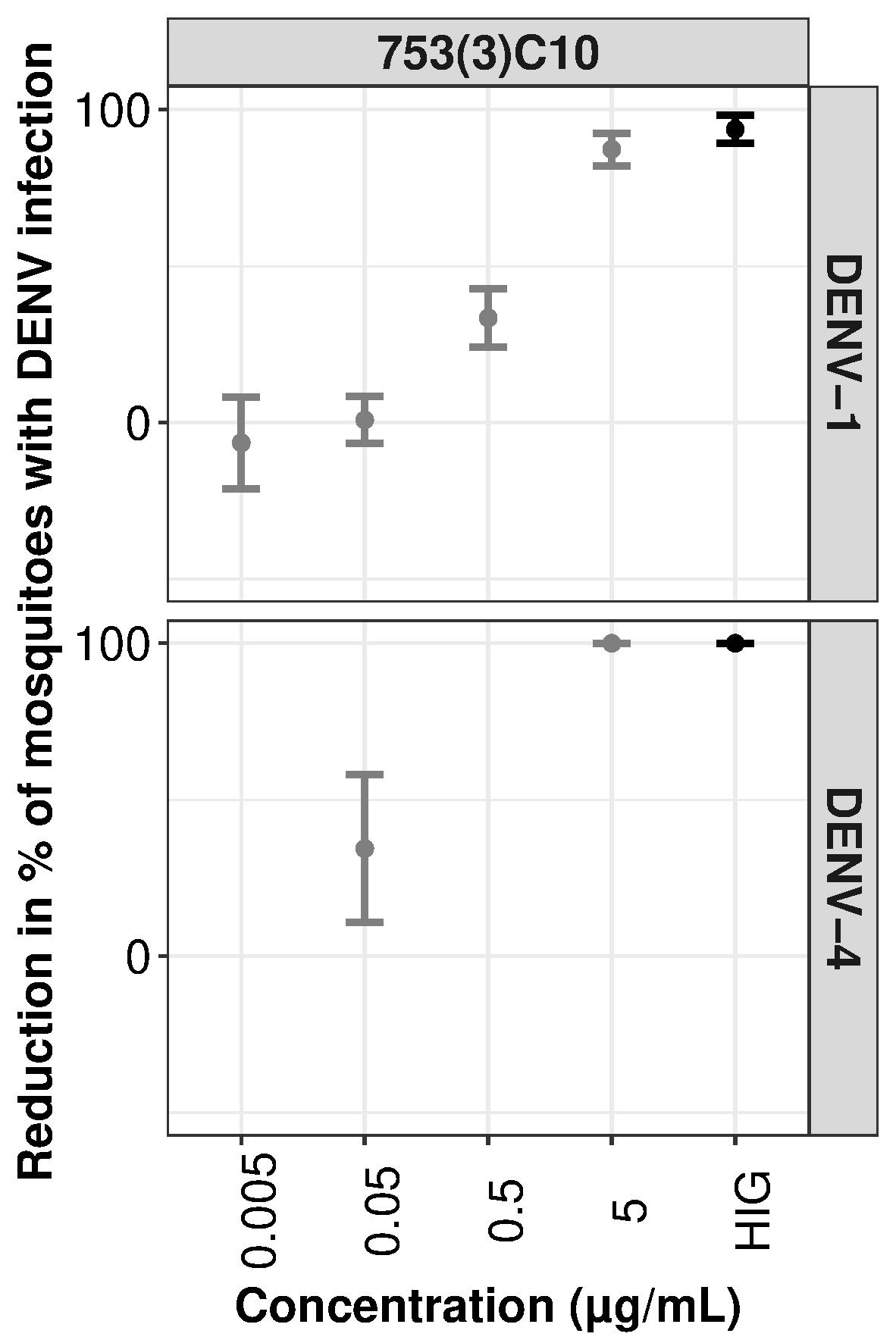

Supplement: S4 Fig — Means and standard errors of more than two replicates are shown. Hyper-immune dengue virus (DENV)-reactive globulin (HIG), used as positive control, is in black while monoclonal antibodies are in gray. The y-axis value of 100 means the mAb blocks DENV infection of mosquitoes completely, relative to the negative control. (TIF) [file pntd.0007142.s004.tif]

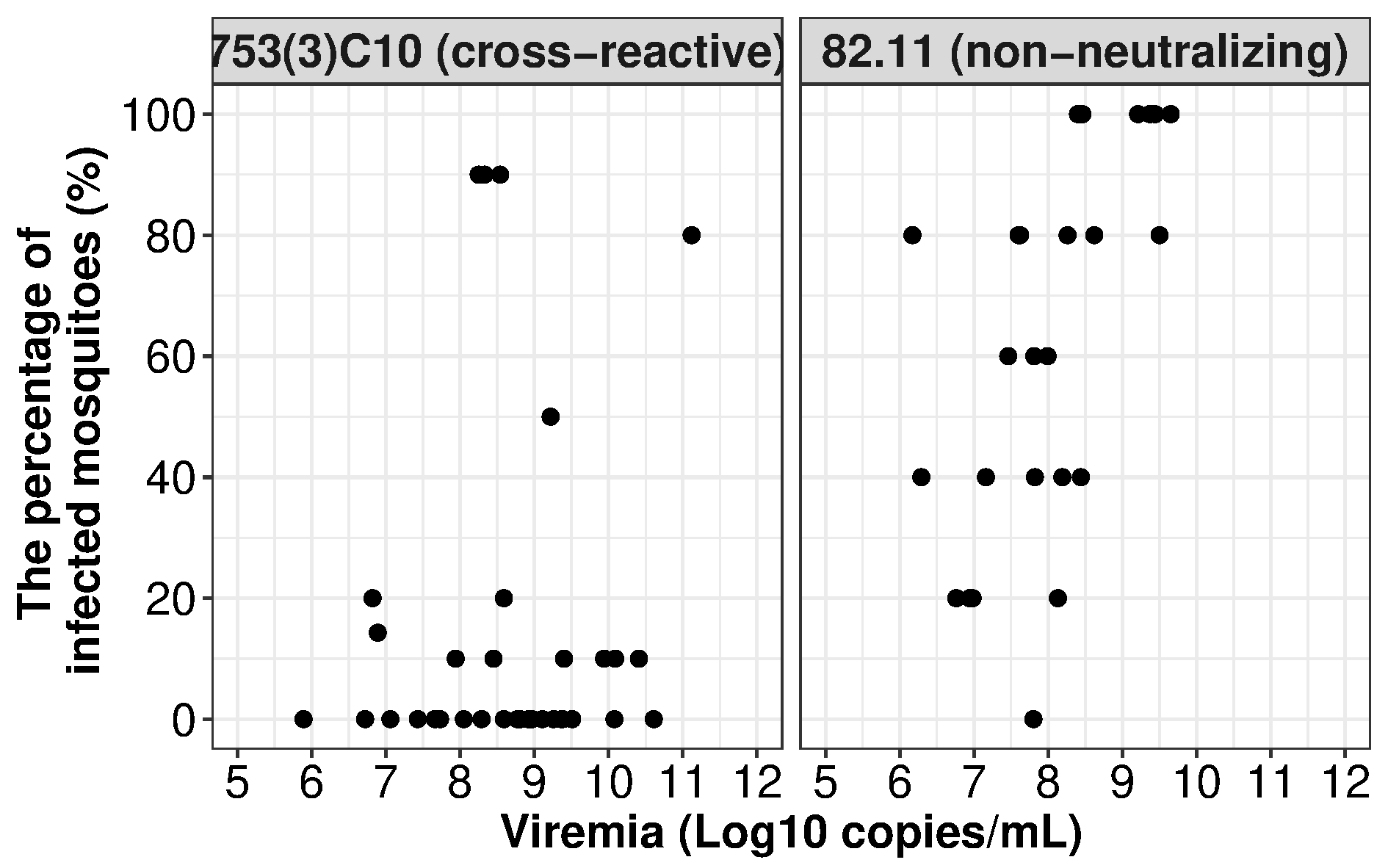

Supplement: S5 Fig — 753(3)C10 and 82.11 were representative of effective and non-effective mAbs, respectively. Each dot represents a single blood meal spiked with the two representative mAbs (10μg/mL). (TIF) [file pntd.0007142.s005.tif]
