# Supplementary material for: Blockade of dengue virus transmission from viremic blood to Aedes aegypti mosquitoes using human monoclonal antibodies
Source: PLoS Negl Trop Dis. 2019 Nov 1;13(11):e0007142. doi: 10.1371/journal.pntd.0007142 (PMC6853333; doi:10.1371/journal.pntd.0007142)
Supplement: S1 Table — Serotype specificity, IC50, and types of epitopes to which these mAbs bind were previously characterized in cited references. Unlike quaternary epitope, linear epitopes consists of individual, separate E proteins. DENV = dengue virus, IC = Inhibitory concentrations. (DOCX) [file pntd.0007142.s006.docx]

| **Clone name** | **Epitope binding type** | **Serotype specificity and IC_50_** | **Reference** |
| --- | --- | --- | --- |
| 1F4 | Quaternary | DENV-1: 0.03 μg/mL | [1] |
| 2D22 | Quaternary | DENV-2: 0.08 μg/mL | [2] |
| 1L12 | Linear | DENV-2: 0.4 μg/mL | [3] |
| 5J7 | Quaternary | DENV-3: 0.56 μg/mL | [4] |
| 1M7 | Linear | DENV-1: 0.55 μg/mL  DENV-2: 0.30 μg/mL  DENV-3: 0.02 μg/mL  DENV-4: 0.50 μg/mL | [5] |
| 1C19 | Linear | DENV-1: 0.06 μg/mL  DENV-2: 0.03 μg/mL  DENV-3: 0.04 μg/mL  DENV-4: 3 μg/mL | [5] |
| 747(4)B7 | Quaternary | DENV-1: 0.10 nM  DENV-2: 0.11 nM  DENV-3: 0.12 nM  DENV-4: 93.19 nM | [6] |
| 753(3)C10 | Quaternary | DENV-1: 0.54 nM  DENV-2: 0.18 nM  DENV-3: 1.89 nM  DENV-4: 0.08 nM | [6] |
| 22.3 | Linear | DENV-4: 0.006 μg/mL | [7] |
| 82.11 | Linear | DENV-1: 0.043 μg/mL  DENV-2: 0.024 μg/mL  DENV-3: 0.090 μg/mL  DENV-4: 0.117 μg/mL | [7] |
| 87.1 | Linear | DENV-1: 0.004 μg/mL  DENV-2: 0.004 μg/mL  DENV-3: 0.008 μg/mL | [7] |
| 14c10 | Quaternary | DENV-1: 1.5 μg/mL | [8] |

1. Fibriansah G, Tan JL, Smith SA, de Alwis AR, Ng TS, Kostyuchenko VA, et al. A potent anti-dengue human antibody preferentially recognizes the conformation of E protein monomers assembled on the virus surface. EMBO Mol Med. 2014;6(3):358-71. doi: 10.1002/emmm.201303404. PubMed PMID: WOS:000332389500007.

2. Fibriansah G, Ibarra KD, Ng TS, Smith SA, Tan JL, Lim XN, et al. Cryo-EM structure of an antibody that neutralizes dengue virus type 2 by locking E protein dimers. Science. 2015;349(6243):88-91. doi: 10.1126/science.aaa8651. PubMed PMID: WOS:000357280800065.

3. Smith SA, de Alwis AR, Kose N, Jadi RS, de Silva AM, Crowe JE. Isolation of Dengue Virus-Specific Memory B Cells with Live Virus Antigen from Human Subjects following Natural Infection Reveals the Presence of Diverse Novel Functional Groups of Antibody Clones. J Virol. 2014;88(21):12233-41. doi: 10.1128/jvi.00247-14. PubMed PMID: WOS:000343314900007.

4. Fibriansah G, Tan JL, Smith SA, de Alwis R, Ng TS, Kostyuchenko VA, et al. A highly potent human antibody neutralizes dengue virus serotype 3 by binding across three surface proteins. Nat Commun. 2015;6:6341. doi: 10.1038/ncomms7341. PubMed PMID: WOS:000350292200001.

5. Smith SA, de Alwis AR, Kose N, Harris E, Ibarra KD, Kahle KM, et al. The Potent and Broadly Neutralizing Human Dengue Virus-Specific Monoclonal Antibody 1C19 Reveals a Unique Cross-Reactive Epitope on the bc Loop of Domain II of the Envelope Protein. Mbio. 2013;4(6). doi: 10.1128/mBio.00873-13. PubMed PMID: WOS:000329174500047.

6. Dejnirattisai W, Wongwiwat W, Supasa S, Zhang XK, Dai XH, Rouvinsky A, et al. A new class of highly potent, broadly neutralizing antibodies isolated from viremic patients infected with dengue virus. Nat Immunol. 2015;16(2):170-7. doi: 10.1038/ni.3058. PubMed PMID: WOS:000348143100009.

7. Beltramello M, Williams KL, Simmons CP, Macagno A, Simonelli L, Quyen NTH, et al. The Human Immune Response to Dengue Virus Is Dominated by Highly Cross-Reactive Antibodies Endowed with Neutralizing and Enhancing Activity. Cell Host Microbe. 2010;8(3):271-83. doi: 10.1016/j.chom.2010.08.007. PubMed PMID: WOS:000282626900008.

8. Teoh EP, Kukkaro P, Teo EW, Lim APC, Tan TT, Yip A, et al. The Structural Basis for Serotype-Specific Neutralization of Dengue Virus by a Human Antibody. Sci Transl Med. 2012;4(139). doi: 10.1126/scitranslmed.3003888. PubMed PMID: WOS:000305629600005.
