# Supplementary material for: Blockade of dengue virus transmission from viremic blood to Aedes aegypti mosquitoes using human monoclonal antibodies
Source: PLoS Negl Trop Dis. 2019 Nov 1;13(11):e0007142. doi: 10.1371/journal.pntd.0007142 (PMC6853333; doi:10.1371/journal.pntd.0007142)
Supplement: S2 Table — Neutralization capacity of 747(4)B7 and 753(3)C10 in the viremic blood neutralization assay, with data for patients-derived DENV-reactive IgG. Mosquitoes were fed on blood meals containing DENV infected blood and 747(4)B7 and 753(3)C10 at 3.7 μg/mL and 5 μg/mL, respectively. Infected mosquitoes were then detected with the presence of DENV RNA. Odds ratios were calculated using data from each mAb with the negative saline control as the reference. P values were calculated using marginal logistic regression models, adjusted for the patient’s plasma viremia and the detection of patients’ DENV-reactive IgG. DENV = dengue virus; PC = positive control; NC = negative control. (DOCX) [file pntd.0007142.s007.docx]

| **mAb** | **Viral**  **Serotype** | **Number of**  **blood meals** | **Percentage of infected mosquitoes**  **(Total number of mosquitoes tested)** | | | **OR**  **(95% CI)** | ***P*** |
| --- | --- | --- | --- | --- | --- | --- | --- |
|  |  |  | **PC** | **mAb** | **NC** |  |  |
| 747(4)B7 | DENV-1 | 20 | 3 (162) | 2 (179) | 86 (170) | 0.00 (0.00-0.01) | **<0.001** |
|  | DENV-2 | 3 | 0 (30) | 63 (30) | 97 (37) | NA^a^ | NA^a^ |
|  | DENV-3 | 0 | NA | NA | NA | NA | NA |
|  | DENV-4 | 6 | 6 (51) | 20 (55) | 76 (75) | 0.07 (0.03-0.20) | **<0.001** |
| 753(3)C10 | DENV-1 | 21 | 3 (170) | 4 (187) | 87 (180) | 0.00 (0.00-0.01) | **<0.001** |
|  | DENV-2 | 3 | 0 (30) | 90 (30) | 97 (37) | NA^a^ | NA^a^ |
|  | DENV-3 | 0 | NA | NA | NA | NA | NA |
|  | DENV-4 | 6 | 6 (51) | 2 (59) | 76 (75) | 0.00 (0.00-0.03) | **<0.001** |

^a^All blood meals were positive with patients’ DENV-reactive IgG, so the model failed.
