# Supplementary material for: Blockade of dengue virus transmission from viremic blood to Aedes aegypti mosquitoes using human monoclonal antibodies
Source: PLoS Negl Trop Dis. 2019 Nov 1;13(11):e0007142. doi: 10.1371/journal.pntd.0007142 (PMC6853333; doi:10.1371/journal.pntd.0007142)
Supplement: S3 Table — (DOCX) [file pntd.0007142.s008.docx]

| **mAbs** | **Serotypes** | **Number of viremic blood meals** | | |
| --- | --- | --- | --- | --- |
|  |  | **DENV IgG positive** | **DENV IgG negative** | **DENV IgG unknown^a^** |
| 747(4)B7 | DENV-1 | 13 | 7 | 2 |
|  | DENV-2 | 3 | 0 | 1 |
|  | DENV-3 | 0 | 0 | 1 |
|  | DENV-4 | 5 | 1 | 3 |
| 753(3)C10 | DENV-1 | 13 | 8 | 2 |
|  | DENV-2 | 3 | 0 | 1 |
|  | DENV-3 | 0 | 0 | 1 |
|  | DENV-4 | 5 | 1 | 3 |

^a^ Untested or inconclusive-IgG samples
